# To sell or not to sell; the differences between regulatory and community demands regarding access to antibiotics in rural Ghana

**DOI:** 10.1186/s40545-018-0158-6

**Published:** 2018-12-14

**Authors:** Samuel Afari-Asiedu, John Kinsman, Ellen Boamah-Kaali, Martha Ali Abdulai, Margaret Gyapong, Osman Sankoh, Marlies Hulscher, Kwaku Poku Asante, Heiman Wertheim

**Affiliations:** 1 Kintampo Health Research Centre, Ghana Health Service, P. O Box 200, Kintampo, Brong Ahafo Region, Ghana; 2 Radboud University Medical Center, Institute for Health Sciences, Radboud University, Nijmegen, Netherlands; 3 Epidemiology and Global Health Unit, Umeå University, Umeå, Sweden; 4 Kintampo Health Research Centre, P.O Box 200, Kintampo, Brong Ahafo Region, Ghana; 5 Centre for Health Policy and Implementation Research, University of Health and Allied Sciences, Ho, Ghana; 6 Statistics Sierra Leone, Freetown, Sierra Leone; 7 Scientific Center for Quality of Healthcare, Radboud University Medical Center, Institute for Health Sciences, Radboud University, Nijmegen, Netherlands

**Keywords:** Antibiotics access, Licensed chemical sellers, Pharmaceutical regulations, Ghana

## Abstract

**Background:**

In Ghana, there is extensive over-the-counter dispensing of antibiotics, resulting in high levels of inappropriate use, and an increase in antibiotic resistance. Regulations prevent Licenced Chemical Sellers (LCS, Over-the-Counter Medicine Sellers) from selling antibiotics other than Cotrimoxazole. In practice, however, these sellers sell a variety of antibiotics. This paper aims to provide insight into the differences between regulatory and community demands on the sale of antibiotics, and to explore how these differences in demand could be resolved to facilitate safe and appropriate use of antibiotics in rural Ghana.

**Methods:**

A total of 32 in-depth interviews were conducted in the Kintampo North and South Districts in Ghana; 16 among antibiotic suppliers, predominantly LCS, and 16 among community members. Six focus group discussions were also conducted among 40 community members. Data were coded using Nvivo 10 and thematically analyzed in line with study objectives. The results are presented as narratives with quotes to illustrate the findings.

**Results:**

Generally, antibiotic suppliers were aware that regulations prevent LCS from selling antibiotics except Cotrimoxazole. However, LCS sell all types of antibiotics because of community demand, economic motivations of LCS, and the poor implementation of regulations that are intended to prevent them from selling these medications. Factors that influence community demand for antibiotics include previous knowledge of effectiveness of some antibiotics, delays in seeking care at health facilities, financial constraints, and distance to health facilities. LCS suggested that they should be trained and allowed to sell some types of antibiotics instead of being prevented completely from selling. Community members also suggested that Community-based Health Planning and Services (CHPS) compounds should be equipped to dispense antibiotics.

**Conclusion:**

The sale of antibiotics by LCS at the community level is influenced by both structural and individual contextual factors. There is a need to educate community members on the appropriate access and use of antibiotics in rural Ghana. In addition, rather than enforcing rules that go against practice, it may be more effective to regulate the sale of antibiotics by LCS and train them to make their dispensing more appropriate. CHPS compound could also be equipped to dispense some antibiotics to improve appropriate antibiotic access at the community level.

## Background

Inappropriate use of antibiotics is a major public health challenge in many Low and Middle Income Countries (LMIC) [1, 2]. In LMICs, there is extensive “over the counter” sale of antibiotics from unlicensed suppliers, resulting in high levels of inappropriate use and a consequent increase in antibiotic resistance (ABR) [3]. This is leading to the loss of many first line antibiotics as effective treatments [4].

One of the major drivers of ABR in LMICs is the existence of plural health systems, whereby both government and private institutions provide health services [5]. Although the existence of multiple healthcare providers increases access to healthcare, there is also a variety of private providers with various degrees of knowledge and approaches to clinical practice, and many of whom are unapproved [6]. As a consequence, people often obtain antibiotics from unauthorized private providers [5]. These sellers may have insufficient training, understanding, and skills, as well as a range of different relationships with formal regulatory systems. At the same time, they are also influenced by financial incentives to sell antibiotics and to comply with customer demands and expectations rather than the law [7].

In Ghana, according to the Health Professions Regulatory Body Act, 2013 (Act 857), only medical doctors, physician assistants, midwives and nurses trained in prescribing are eligible to prescribe registered antibiotics [8]. Moreover, the Pharmacy Act, 1994 (Act 489) includes relevant sections on the dispensing and sale of medicines, including antibiotics. According to section 31 of the Act, ‘*no person shall do a business of supplying from any premises restricted drugs classified by regulations as class A drugs/prescription only medicines, class B drugs/pharmacy only medicines or class C drugs/over the counter medicines, unless that person has a valid general or limited license’* [9]. About 80% of medicine outlets in rural communities in Ghana are Licenced Chemical Sellers (LCS, over-the-counter medicine sellers), who are mostly the first point of contact for healthcare [10]. Section 29 of the Pharmacy Act, explicitly prevents these LCS from selling class A and B medicines including antibiotics [9], except for oral Cotrimoxazole which is usually in suspension and commonly dispensed for the treatment of infective diarrhea, urinary tract infections and upper respiratory tract infections [11].

In line with these regulations, pharmacies are allowed to dispense Amoxicillin, Flucloxacillin, Norfloxacin + Tinidazole, Ciprofloxacin, Doxycycline, Tetracycline, Erythromycin and Ampicillin based on the recommendation of a practicing pharmacist [8, 12]. However, beyond the authorized antibiotics, pharmacies and LCS frequently dispense other antibiotics with or without prescription. To secure, in the public interest, the highest standards in pharmacy practice, a regulatory body - the Ghana Pharmacy Council - was established, but it has inadequate resources to fulfil its mandate [13].

Considering the influence of regulation and community (including LCS and community members) demand on access to antibiotics, interventions aimed at addressing the accessibility challenge need to consider synthesizing the relationships between regulatory structures and the community [6, 14]. Interventions including regulations that take into account the demands of the community are more likely to be successful [14, 15]. This paper aims to provide insight into the differences between regulatory and community demands on the sale of antibiotics, and to explore how these differences in demand could be resolved to facilitate safe and appropriate use of antibiotics in rural Ghana.

### Theoretical perspective

This study topic, concerning the differences in demand between regulatory structures and the community on the sale of antibiotics in Ghana, can be examined through the lens of the theory of structuration. Structuration is a social theory concerning the production and reproduction of social systems that is based on the analysis of both *social structures (social forces/institutions)* and *agents (individuals)*, but without giving primacy to either [16–18]. It explores the extent to which, and how social forces and individuals shape our social reality. ‘Structures’ are rules and resources that individuals draw upon in their activities/practices and that produce and reproduce social systems. Social structures influence, for example, the economic system, legal system, health system, political system, and culture. ‘Agency’ refers to the capacity of individuals to act independently of these structures and to make their own free choices. Thus individuals have the ability to monitor and evaluate their actions in the context within which these actions take place [16–19].

It is important to note that the relationship between structures and individuals is bi-directional. The structure-agency relationship as outlined in structuration theory implies that people ‘make’ society, but are at the same time they are constrained by it. Consequently, structures and actions of individuals cannot be analysed separately [16].

Situating our context within structuration theory, the antibiotic regulatory structures in Ghana (Pharmacy act, Pharmacy council) and human agency (LCS and community members) are not mutually independent, but rather they comprise two complementary components of the framework that determines the sale of medicines, including antibiotics. This theory can help us to understand the intersection of practices in structural relations and how institutionalized individual practices connect to produce system integration.

## Methods

The methods for this study are reported according to the consolidated criteria for reporting qualitative research framework (COREQ) [20].

### Study design

This study is part of the Antibiotic Access and Use (ABACUS) study, which has employed a mixed methods approach among antibiotic suppliers and community members across six Low and Middle Income Countries in Asia and Africa, including Ghana [21]. This paper reports on factors affecting community antibiotic access and use that prevailed in the study area that were explored through qualitative in-depth interviews and Focus Group Discussions (FGDs).

### Study area

Data were collected in the Kintampo North and South Districts in Ghana between January and June, 2017. The two districts are located within the forest-savannah transitional ecological zone in the Brong-Ahafo Region. The study area covers an area of 7162 km^2^ with a resident population in 2013 of approximately 151,000 [22, 23]. The study setting is largely rural and subsistence farming is the major occupation. The majority of inhabitants in this area initiate treatment for some ailments at home, then, if necessary, continue to the LCS to buy medicines, including antibiotics. They may finally end up in a public health facility if their illnesses do not resolve [24]. Public health facilities (government owned) in the area include two hospitals, 12 health centres/ clinics, and 30 Community-based Health Planning and Services (CHPS) compounds; while the privately owned health facilities include four clinics, two maternity homes, four pharmacies, and 86 LCS. In addition to these formal private and public health providers, there are also informal medicines sellers who provide health services to urban as well as to the majority of deprived rural poor community members in the study area.

#### Data collection

Issues on the differences in regulatory and community demands on the sale of antibiotics, and on how the differences in demand could be resolved were explored through qualitative in-depth interviews (IDIs) and Focus Group Discussions (FGDs). IDIs were used to collect information about individual respondents’ experiences and opinions, while the FGDs were used to derive insights into community norms on the topic. Antibiotic supplier and community member IDIs and community member FGDs were performed consecutively; themes emerging from the IDIs were used to inform the discussions in the FGDs.

### Selection of participants

- *Antibiotic suppliers IDIs*

Sixteen IDIs were conducted among dispensers of antibiotics including two pharmacists, two dispensing technicians, one physician assistant, one health assistant, three community health officers, and seven LCS (Table 1). The respondents were broadly representative of the categories of antibiotic dispensers in the study area: all purchase or dispensing points for antibiotics in the study area (public or private, from public hospital pharmacies to street vendors) had previously been identified and mapped. Antibiotic suppliers were eligible for IDIs if they were 18 years or older and if they were dispensing or selling antibiotics as observed through the mapping exercise. The suppliers were purposively selected to include similar proportions of supplier types identified in the mapping exercise.

- *Community members IDIs*

**Table 1:** Summary of interviews and categories of respondents for IDIs and FGDs

Type of interview and respondents categories	No. of respondents/participants
Antibiotic suppliers IDIs	
Pharmacists	2
Dispensing Technicians	2
Physician Assistant	1
Health Assistant	1
Community Health Officers	3
LCS	7
Community members IDI	
Mothers of children ≤5 years	8
Males ≥18 and < 60 years	2
Females ≥18 and < 60 years	2
Males ≥60 years	2
Females ≥60 years	2
Community member FGDs	
Females ≥18 and < 30 years old	6
Males ≥18 and < 30 years old	8
Females ≥30 years old	6
Males ≥30 years old	6
Female church leaders ≥18 years old	8
Male primary school teacher ≥18 years old	6
Total	72

Community members who were 18 years old and above were randomly selected from the database of the Kintampo Health and Demographic Surveillance for the study area [22]. The database contains a list of all residents in the study area. Selected community members who were willing to speak about their experiences with and attitudes towards medicines were interviewed. All community members who were selected agreed to be interviewed. Sixteen IDIs were conducted among community members from different households. The 16 IDIs were conducted with eight mothers who care for children five years or younger and eight community members (two males and two females younger than 60 years, two males and two females of 60 years or older) (Table 1).

- *Community members FGDs*

Six FGDs were conducted among community members to further discuss community norms on access to and use of antibiotics. In the first four FGDs, community members 18 years old and above were randomly selected from the HDSS database to participate in this study. Each group was made up of 6–8 community members. The participants in the other two focus groups (female church leaders and male primary school teachers) were purposively selected as social groupings relevant to the local context (Table 1).

### Data collection procedures

Eligible antibiotic suppliers and community members were informed about the purpose and procedures of the study. The written information sheet as well as the informed consent criteria were read to the potential participants. Participants who consented for the interviews were provided with the copies of the written study information sheets, and signed inform consent forms were collected before the actual interviews were carried out. Each discussion session was audio recorded and conducted by a moderator and a note-taker. Community members IDIs and FGDs were held in Twi dialect (a widely spoken local dialect) in the compound of the participant, a church, classroom, or under a shaded open space. Antibiotic suppliers IDIs were conducted in Twi or English in the premises where the medicines are sold to clients at a time when attendance was very low to avoid interruption by potential clients. The discussions were facilitated by a moderator using an interview guide that was made up of predetermined questions and themes. The main themes explored include regulatory and community demands on the sale of antibiotics and how the differences in demand could be resolved to improve appropriate use of antibiotics. Other related emerging issues were also discussed. Notes on responses and other non-verbal communications were also taken by a note-taker. IDIs and FGDs generally lasted for about 30 min and one hour respectively. Interview sessions were brought to an end when the moderator had exhausted all questions on the interview guide and on other emerging issues.

### Data management and analyses

A thematic analytical approach was used in the management and analysis of the qualitative data. The processes used for the analysis followed the approach proposed by Braun and Clarke [25]. The audio recordings of the interviews were transcribed into English verbatim by the researchers. Interviews conducted in Twi were translated into English during transcription. Transcripts were then checked for completeness and accuracy by vetting them to match the audio recordings, whilst we familiarized ourselves with the data to gain a broad understanding of the content of the interaction and whilst also taking notes of important ideas. The transcripts were imported into NVivo10. In the NVivo, a priori themes were developed around regulatory and community demands on the sale of antibiotics and on how the differences in demand could be resolved to guide the coding of transcripts. During the process, more themes and sub-themes emerged that captured other, inductively-derived issues. This was followed by interpretive analysis of the collated codes whereby themes were combined, refined, separated or discarded where necessary.

## Results

The Results section is made up of four sub-sections, including (i) demographic characteristics of the respondents, (ii) the regulatory demands on the sale of antibiotics, (iii) community demands for antibiotics, and (iv) suggestions on how to resolve the differences in regulatory and community demands on the sale of antibiotics. Results are presented as a narrative with selected quotes to support the findings.

**(i) Demographic characteristics of respondents.**

A total of 72 respondents participated in the study of whom 28 (39%) were males and 44 (61%) were females. Sixty percent of the respondents were between the ages of 18 to 35 years. Also, 26 (36%) of the respondents had no formal education, and 23 (32%) were farmers.

**(ii) Regulatory demands: knowledge of the regulations governing the sale of antibiotics.**

It was common knowledge among all categories of suppliers that antibiotics are supposed to be dispensed by hospitals and pharmacies according to regulations:

*There are categories of facilities that can sell or dispense antibiotics. When you come to the hospital we can dispense antibiotics, but in the communities I know only accredited pharmacies can sell and dispense antibiotics. We have LCS but they are not supposed to sell antibiotics* (IDI, Pharmacist#2).

Whilst this finding was corroborated by some LCSs, it also emerged that others are unaware that they are permitted to sell cotrimoxazole.

*The license I am using in this shop right now is class C, thus for the Licenced Chemical Seller to sell pain killers and some anti-histamines… let’s say something like first aid. So we are only dealing with first aid for the meantime. That is what we have been licensed to be selling. In my case as a chemical seller, I am not supposed to dispense any antibiotic. In no case should I dispense any antibiotic* (IDI, LCS #1).

There were definite responses on the categories of practitioners who are approved to dispense antibiotics:

*A pharmacist can dispense antibiotics, a doctor can dispense, a dispensary technician, [but a] Licenced Chemical Seller cannot dispense, and sometimes the midwives. Even nurses are not allowed to dispense or give antibiotics. Apart from these people, anybody selling or dispensing antibiotics is illegal* (IDI, LCS #1).

Generally, dispensers mentioned that antibiotics are prescription-only medicines that are to be dispensed by only approved and qualified practitioners.

*Antibiotics are prescription-only medicines. You don’t “use an oral means” (make a verbal request) to buy. The dispenser must therefore be professional to understand that they can be sold with prescription only (IDI, Pharmacist#1).*

By contrast, community members were generally unaware of any regulations governing the sale of antibiotics. It emerged they do not know that they are not supposed to buy antibiotics from the LCS, except cotrimoxazole.

*We have not heard anything that if you want to buy antibiotics, you have to see a doctor first or buy from the Pharmacy. We think once it is in the Licenced Chemical Seller’s shop, you can go and buy* (IDI, mother of under-five#1).

**(iii) Community demands for antibiotics.**

In spite of the regulations, dispensers and community members mentioned that antibiotics can be accessed from all types of medicine sellers and dispensers at the community level, with or without prescription:

*Yes, I can easily buy it without prescription. Especially if I know it is good for me. Yes, recently I bought the white antibiotics [Chloramphenicol] I told the supplier that I needed some for my son’s sore. They did not say anything, neither did I ask any questions* (IDI with Community members, Females 18–30 years, respondent#1).

*Most of the antibiotics we take are not prescribed by a Doctor; we just buy and take it like that. For instance, when someone has stomach ache, he just buys red and yellow (tetracycline) and pours it into akpeteshi [locally brewed gin from palm tree] and takes* (FGD, Primary school teachers respondent#5).

This finding was corroborated by suppliers:

*Yes, they come to ask; for the medicines we sell here, they come to ask for specific medicine and we give it to them. Over here it is usually, I am buying this, I am buying that* (IDI, LCS #2).

*They ask for the Amoxicillin, Ampicillin and Flagyl. When they come they say this is what I want, they will not explain to you that I am buying this because this is what is happening to me. They say what they want and you give it to them* (IDI, LCS #4).

A variety of reasons were given for community members accessing antibiotics without prescription, as explained below.

- ***Previous knowledge and experience***

Community members who have previously experienced the effectiveness of some antibiotics tend to seek out the same antibiotics when they are sick, or they may recommend such antibiotics to others who may be experiencing similar symptoms to their own.

*Many of them ask for particular drugs, sometimes based on recommendations from others on the effectiveness of the drugs* (IDI, Dispensing Technician #1).

*We have a problem with that. Some patients come with some packages of drugs they have used before and ask for those drugs, even if the condition is not related to the drug they are asking for* (IDI, Physician Assistant).

*Others also come to point to what they want. They say this is what I usually take, this is what the doctor normally prescribes for me and it works for me* (IDI, LCS #3).

This finding was confirmed by at least one community member:

*If you have previously used an antibiotic, you just go and buy one from the drug store. Recently I bought some antibiotics (Amoxicillin) because I knew what I wanted [so] I just went to the drug store and asked for it.* (IDI, Female community member_18-60 years, respondent #1).

b) ***Delays at hospital***

Delays in receiving treatment at the hospital constituted a major disincentive for seeking care at the hospital as well as an incentive for people to seek care at the LCS shops and other medicine sellers. This issue was expressed by both suppliers and community members:

*Yes, self-medication is very common and it is the fault of the hospital. If someone goes to the hospital, they can stay there the whole day [before they are seen by a doctor] - that is not good. So people are reluctant to go to the hospital for malaria and cough* (IDI, Dispensing Technician#2).

*I will say something about the hospital; a lot of people don’t like going to the hospital. What they say is that when they visit the hospital, they will sit there for a very long time before they attend to them, so you may end up dying with the illness you took to the hospital. For this reason, they will go to the drug store because they know when they buy the medicine it will cure their illness; so they will move straight to the drug store to buy medicine to cure their illness because they will waste their time when they go the hospital* (FGD, female church leaders, respondent#8)

c) ***Financial challenges***

Financial constraints also came up in the responses as one of the major reasons for accessing antibiotics from unapproved medicine providers, without prescription.

*Some people go to drug stores to buy medicine when they are sick because they think that when they go to the hospital they can’t pay, and the transport too is a challenge* (FGD male above 30 years, respondent#4).

Related to the cost is the fact that medicines are usually prescribed in the hospital for people to go and buy in the open market, so it is better for them to go and buy from the drug shops where they think medicines are relatively cheaper.

*In terms of cost, some people say that when they go to the hospital, medicines will be prescribed for them to go and buy from the open market, so there is no need to go to the hospital… it is better they enter the drug stores themselves* (FGD, Primary school teacher, respondent#2).

*The money you spend buying medicine from the drug store is less than what you spend at the hospital.* (FGD, female church leaders, respondent #2)

d) ***Distance to health facilities***

Long distances to the nearest health facility was a challenge especially when the illness is perceived to be unserious.

*Distance is another challenge because some people stay far away from health facilities so when they are sick it becomes a big challenge for them to reach the facilities.* (FGD male above 30 years, respondent#3)

e) ***Financial gains by LCS***

Dispensers in the community who are mostly LCS are not prepared to stop selling antibiotics because of the money they make from them, even though they know it is against regulations. The disposition by LCS to continue the sale of antibiotic was because stopping the sale of antibiotics will affect their business:

*The other drugs do not run [get sold] fast. They just come to buy ten pesewas, 20 pesewas (< 0.05USD)* etc. *How long will it take to recover your money? But with the antibiotic, they buy GHC 4 in Ghana currency (0.91USD), GHC 3 (0.68USD), so even if they buy 10 a day, you make something out of it.* (IDI, LCS #3)

f) ***Weak implementation of regulations on antibiotic sales***

It also emerged that the regulation regarding the sale of antibiotics is weakly implemented by Pharmacy Council. LCS and drug peddlers therefore evade the regulations concerning antibiotics.

*The checks are not done well…. Let’s say if he (pharmacy council person) should come here today, the very moment he gets to town all the chemical sellers around will get to know the pharmacy council person is in town and they will all hide their things [antibiotics].* (IDI, LCS #1).

*Though the regulations governing the sale of antibiotics are sufficient, we always find a way around it* (IDI, LCS #1).

*So whether they train us or not, we will sell. We will hide to sell* (IDI, LCS #3).

**(iv) Suggestions on how to resolve the differences in regulatory and community demands on the sale of antibiotics.**

Generally, dispensers mentioned that instead of preventing LCS from selling antibiotics, they should rather be trained to sell antibiotics in a proper, safe way. This is because there is a demand for antibiotics at the community level while there are no approved dispensers of antibiotics there or other good access to healthcare:

*We want the doctors to train us so that we can sell to support the community, because it is not everyone who can go to the hospital in town. So if they train us to know why we are not allowed to sell, then we can know [what to do] to be able to support them. When the malaria Rapid Diagnostic Test kit was introduced, we were trained on how to do the test and we are able to do that. Initially we treated all fever cases as malaria and people continued to take the antimalarial.* (IDI, LCS #3).

To facilitate the appropriate dispensing of antibiotics by the LCS, it was suggested that antibiotics are rather packaged as complete doses for specific disease episode (as opposed to current practice whereby individual pills are placed into a small paper bag by the seller) and dispensed according to treatment guidelines.

*I think the antibiotics should come as complete packs, so that lower cadres like us can also dispense.* (IDI, LCS #1).

*I was trying to talk about treatment guidelines. For all the medicine we are using, we have treatment guidelines so if the antibiotic that you are giving is not in that treatment guideline, I don’t think we can use it*. (IDI, Health Assistant #1).

Some community members also suggested that the government should equip the Community Based Health and Planning Services (CHPS) compounds with the capacity to dispense antibiotics. CHPS is a community-based approach for extending health services to deprived communities through community-based service delivery point (a ‘CHPS compound’). These are managed by Community Health Officers who provide clinical care (including basic antibiotic prescriptions) for minor ailments as well as preventive and health promotion services delivered through house-to-house visits.

*What I think is that, we should furnish the CHPS compounds with the necessary equipment and personnel as we do for the bigger hospitals. This will be helpful so that if even the person has no money to travel to the bigger hospital, at least they can walk to the CHPS compound*. (FGD, Males 18-30 years respondent#8).

## Discussion

This paper reports on the differences in regulatory and community demands on the sale of antibiotics in rural Ghana. The research was conducted in a context where private health facilities such as Licenced Chemical Sellers (LCS), who are not permitted to sell antibiotics, contribute significantly to the provision of healthcare, including through the illegal sale of these medicines.

In Ghana, a wide range of antibiotics is available on the open market, and acquiring drugs over the counter is a very common practice [26]. This is in spite of the Pharmacy Act, 1994 (Act 489), which prevents LCS from selling and dispensing Class A/prescription only medicines, and B Class /pharmacy-only medicines including antibiotics. Our findings of unregulated access to antibiotics without prescription reflects the results of studies in other LMICs [1, 3]. In some higher income countries like Spain, the practices of un-prescribed antibiotics dispensing is also reported to be on the increase [27–30]. In line with the theory of structuration the findings in our study and from others indicate that a new form of structure regarding access and sale of antibiotics has been created informally, to serve the needs of LCS (or their equivalent) and community members. This practice requires urgent attention in order to maintain the effectiveness of antibiotics [16, 17, 31].

The sale of antibiotics by LCS with or without prescription is as a result of weak implementation of regulations on antibiotic sales, along with prevailing community customer demand for antibiotics and financial gain by LCS. As in other studies conducted in LMICs [27], the most common reasons given for selling antibiotics without prescription is customer demand. Consequently, over-the-counter medicine sellers stock and sell antibiotics against regulations in order to meet the demands of their customers and to maximize profit [27]. Customers insist on buying antibiotics even if dispensers think that the antibiotics they are asking for may not be right for their conditions [32]. We found that some customers insist on certain antibiotics because of previous use and knowledge of their effectiveness. This shows that LCS and community members may be engaging in these practices because they lack sufficient knowledge and information to understand the risks of their behaviours in which case public health education is key. Also comparable to other studies, community members’ persistent interest in buying antibiotics without prescription from LCS was the result of their being relatively cheaper than the cost from the pharmacy or the cost of attending hospitals for treatment [27]. It is important to recognise, however, that access to antibiotics from LCS occurs not only because of the weakness of the regulation on the sale of antibiotics. As the structuration theory indicates, it is also as a result of the fact that human agency (manifested through LCS and community members) and social structures (manifested through regulations on antibiotics) have different demands with regards to the sale of antibiotics.

Community members also demand and buy antibiotics without prescription from LCS because of the delays found in health facilities. It is therefore important to improve the health system whereby waiting times are reduced because, as per the principle of social structure reproduction, if individuals find it difficult to act in any way that is expected, social forces will emerge that provide incentives to act otherwise [16, 17, 31]. For instance delays in accessing health care in hospitals can push individuals to demand for and buy antibiotics from LCS who are close to them in their communities and other unapproved sellers [33]. Our findings on delays in hospitals is comparable to study results from Kenya and Sudan, where 45 and 39% of respondents respectively said they self-medicate because of delays in hospitals [34, 35]. Closely linked to the delay is the distance to appropriate antibiotic sales points/dispensers. This encourages people in some communities to purchase antibiotics from LCS in the case of Ghana, without prescription. Similar to this finding, a study in India found that participants who had no access to a licensed allopathic trained doctor in their own village, and who faced significant travel costs, often made the decision to seek care directly from unapproved dispensers in their villages [36]. We also found that a lack of knowledge about the approved dispensers of antibiotics by customers is a reason for buying antibiotics without prescription. This is because customers perceive dispensers in the community as professionals, and they trust them [4].

The overarching goal of improving antibiotic access and use therefore partly hinges on resolving the differences in demands between the regulatory authority and community members on access to antibiotics. Resolving the differences in demands will require a collaborative approach among the Ghana Pharmacy Council, the LCS Association of Ghana, the Ministry of Health, and other stakeholders.

To resolve the differences in demands, LCS suggested that, considering the unmet needs in Ghana’s healthcare system, they should be trained and permitted to sell some antibiotics, instead of being prevented completely from the sales of antibiotics. They argue that the creation of a system which synthesises the demands of the regulations with those of community members and themselves will facilitate safe and appropriate antibiotic access and use. Thus there is an opportunity for Ghana to develop an innovative approach by restructuring its regulatory framework and to train LCS, which could also be a model for other countries struggling with inappropriate antibiotic sales. A systematic review of literature could provide further evidence on the successes or failures of intervention studies which aimed to improve on the dispensing practices of Over-the-Counter Medicine Sellers in similar settings. However, as shown by studies in Vietnam, training of drug sellers in private pharmacies can be effective in changing their knowledge and dispensing practice [37, 38]. LCS in Ghana could therefore be trained to dispense WHO “key access antibiotics” (first or second choice antibiotics). According to WHO, these antibiotics should be widely available, affordable and quality assured to improve antibiotic access and health outcomes. Training LCS is tandem with WHO 2017 expert committees’ recommendation that “key access antibiotics” should be a subject of targeted or specific stewardship programmes [39].

Leveraging the training of LCS is in line with the Pharmacy Councils’ mission of collaborating with relevant stakeholders (in this case the LCS) to enhance their effectiveness and contribution to appropriate drug use in Ghana [13]. This should also be seen against the background in which the regulators (i.e. the Pharmacy Council) lack adequate resources to be able to monitor and enforce the regulations on the sales of antibiotics. As a result, suppliers hide the antibiotics that they hold in stock when regulators go for inspection. The task-shifting concept of training Licence Chemical sellers to dispense some categories of antibiotics may be seen as viable, because these same sellers in Ghana have previously been trained to test their clients with malaria symptoms by using Malaria Rapid Diagnostic Test kits before dispensing malaria medicines [40, 41].

Another possible point of entry for resolving the problem could come through the CHPS compounds in communities, which could be equipped to dispense some categories of antibiotics. CHPS is a national primary health care programme designed to remove geographic barriers to health care, and thereby to bridge the gaps and inequality in healthcare provision. It is a structure for the realization of the goals of primary health care and the program of work for the Health Sector Reforms for Ghana Vision 2020 [42]. The call for Community Health Officers in CHPS to be trained to dispense antibiotics reiterates the high emerging demand of the community for curative services provision in the CHPS facilities, as specified in the revised CHPS policy launched in 2016 [42]. As a study in Vietnam indicates, equipping healthcare providers at community level could improve their dispensing of antimicrobials [38]. This suggestion could be facilitated by strengthening the clinical orientation on the treatment of minor ailments such as malaria, diarrhoea, acute respiratory infection given to Community Health Officers post-graduation before they are posted to the CHPS compounds.

The findings from this study reflect the principles of the theory of structuration, which explores the relationship between individuals and the social structures that shape our social reality. Our findings indicate that while there may be established social structures in the form of traditions, institutions, moral codes and ways of doing things, these structures can be changed when people ignore them, replace them, or reproduce them differently. In our context, regulations prevent LCS from legally selling antibiotics beyond cotrimoxazole, but this structure has changed because LCS and community members have found it to be inadequate to their respective needs, and thus they have ignored and replaced it with a new system whereby all types of antibiotic are sold by LCS.

### Limitation and the need for further studies

This study has explored the perspective of suppliers and community members on the differences between regulatory and community demands on the sale of antibiotics, and how the differences in demand could be resolved to improve on appropriate access, and use of antibiotics. The views of policy makers are not represented in this paper. Further studies will be required to explore the perspective of policy makers for a holistic view on how to meet the demand for antibiotics at the community levels in Ghana while simultaneously ensuring that access to and use of these drugs is optimized and safe.

### Conclusion and recommendation

Access to antibiotics at the community level is influenced by an interplay of structural and individual contextual factors. At the structural level, regulations are in place to restrict important community-level suppliers of medicines, such as LCS, from selling antibiotics (apart from cotrimoxazole); however, at the community level, antibiotics are sold because the community demands them. The Pharmacy Council and other relevant stakeholders are encouraged to consider training sellers at the community level to dispense some essential antibiotics in a manner that will optimise their safe use. Without such a change in the regulations, LCS will continue to hide and sell antibiotics, thereby promoting unsafe use of antibiotics and, ultimately, resistance to the drugs. Training LCS to dispense antibiotics would comprise a collaborative and contextualized approach that would synthesise the differences in demands of regulation and the community concerning access to antibiotics. This approach will also help the Pharmacy Council to achieve its mission of securing the highest level of pharmaceutical care in Ghana, while ensuring the availability of competent pharmaceutical care providers who practice within agreed standards and who are accessible to the whole population. At the same time, the Ministry of Health/Ghana Health Service may also want to consider equipping CHPS compounds so that they are able to dispense antibiotics, thereby facilitating appropriate access to antibiotics.

## Abbreviation

ABACUS: Antibiotic Access and Use
CHPS: Community-based Health Planning and Services
FGD: Focus Group Discussions
IDI: In-Depth Interviews
KHRC: Kintampo Health Research Centre
LCS: Licensed Chemical Seller/s
